# Alcohol as an unproductive strategy for coping with psychological distress in depressive disorders

**DOI:** 10.1192/j.eurpsy.2026.10894

**Published:** 2026-07-31

**Authors:** T. I. Medvedeva, S. N. Enikolopov, O. Y. Vorontsova, O. M. Boyko, M. I. Oleychik

**Affiliations:** clinical psychology, Federal State Budgetary Scientific Institution Mental health scientific center, Moscow, Russian Federation

## Abstract

**Introduction:**

Alcoholism is quite often combined with other mental illnesses. There are two aspects of the interaction between alcoholism and mental disorder: the influence of mental disorder on alcoholism, and the possible influence of alcohol on mental disorder.

**Objectives:**

The aim of the study was to analyse the specifics of alcohol use by patients with depression, assessment of the connection with ego identity.

**Methods:**

77 patients (31.2% male, mean age 21.8±4.3) with depressive states (F31.3-4; F34.0; F21.3-4+F31.3-4; F60.X+F31.3-4).

Alcohol consumption was assessed using the ‘alcohol consumption’ scale of The questionnaire for diagnosing manifestations of psychological distress (Rasskazova, Sadovnichaja, 2022) was developed for research by E.G. Demenko (Demenko et al., 2018). Based on this scale, the subjects were divided into two subgroups: ‘No alcohol consumption’ (N=24) and ‘Alcohol consumption’ (N=53), that did not differ in terms of socio-demographic indicators.

SCL-90-R, Cambridge Depersonalisation Scale (CDS), Ammon Ich Structur Test (ISTA) measures personality traits formed as a result of early relationships with significant others, Constructive Thinking Index (CTI), Questions about the propensity for risky behavior (answers on a Likert scale) were used. Subgroups were compared using analysis of variance in SPSS.

**Results:**

The results showed that the group ‘Alcohol consumption’ had higher scores on the SCL-90R scales, dissociative phenomenology (CDS), categorical thinking (*СТI*), high level of destructive internal and external ego demarcation (ISTA) (see Tabl.). The ‘Alcohol consumption’ group also has a higher level of risk-taking behaviour (20% in the no-alcohol group versus 50% in the alcohol group).

**Table 1.** Comparison of subgroups without and with alcohol consumption Table 1. Long description.

	No alcohol consumption (N=24)	Alcohol consumption (N=53)	Sig.
SCL–90R GSI Global Severity Index	1,112±0,585	1,602±0,653	,002
SCL–90R PSI Positive Symptom Total	45,750±17,718	59,415±18,170	,003
ISTA Destructive Aggression	55,283±8,809	62,174±9,755	,004
ISTA Destructive Anxiety/Fear	68,965±11,990	75,521±12,600	,036
ISTA Destructive Outer ego demarcation	55,343±14,897	63,483±12,071	,013
ISTA Destructive Inner ego demarcation	53,717±8,694	62,886±9,249	,000
CDS _ASR	10,813±8,773	16,250±8,851	,039
*СТI* _Categorical Thinking	34,606±6,932	41,155±10,008	,001

**Conclusions:**

Alcohol consumption in mental illnesses is associated with higher rates of psychopathological symptoms, categorical thinking, and a tendency to risky behaviour. The data obtained suggest that a significant role is played by EGO- integration disturbance (rigid boundaries, destructive fear, pathological narcissism, destructive aggression), which are associated with higher rates of the alcohol consumption. Alcohol can be considered as a vulnerability factor for mental disorder, and alcohol can be used as an unproductive coping strategy in cases of severe psychological distress.

**Disclosure of Interest:**

None Declared

## Long descriptions

### Table 1. Long description

The table consists of four columns: Metric, No alcohol consumption (N equals 24), Alcohol consumption (N equals 53), and Significance (Sig.). Values are presented as mean plus or minus standard deviation.

* S C L 90 R G S I Global Severity Index: No alcohol 1,112 plus or minus 0,585; Alcohol 1,602 plus or minus 0,653; Sig. ,002.

* S C L 90 R P S I Positive Symptom Total: No alcohol 45,750 plus or minus 17,718; Alcohol 59,415 plus or minus 18,170; Sig. ,003.

* I S T A Destructive Aggression: No alcohol 55,283 plus or minus 8,809; Alcohol 62,174 plus or minus 9,755; Sig. ,004.

* I S T A Destructive Anxiety/Fear: No alcohol 68,965 plus or minus 11,990; Alcohol 75,521 plus or minus 12,600; Sig. ,036.

* I S T A Destructive Outer ego demarcation: No alcohol 55,343 plus or minus 14,897; Alcohol 63,483 plus or minus 12,071; Sig. ,013.

* I S T A Destructive Inner ego demarcation: No alcohol 53,717 plus or minus 8,694; Alcohol 62,886 plus or minus 9,249; Sig. ,000.

* C D S _ A S R: No alcohol 10,813 plus or minus 8,773; Alcohol 16,250 plus or minus 8,851; Sig. ,039.

* C T I _ Categorical Thinking: No alcohol 34,606 plus or minus 6,932; Alcohol 41,155 plus or minus 10,008; Sig. ,001.

Navigate back to Table 1..

